# Schneiderian membrane thickness variation following endodontic procedures: a retrospective cone beam computed tomography study

**DOI:** 10.1186/s12903-020-01122-6

**Published:** 2020-05-06

**Authors:** Thera Van Den Munckhof, Shanon Patel, Garrit Koller, Erwin Berkhout, Francesco Mannocci, Federico Foschi

**Affiliations:** 1 Department of Oral Radiology, Academic Centre for Dentistry Amsterdam, ACTA, Gustav Mahlerlaan 3004, 1081 LA Amsterdam, The Netherlands; 2 Department of Endodontics, Faculty of Dentistry, Oral & Craniofacial Sciences, King’s College London, Guy’s Hospital, Tower Wing, Floor 22, London, SE1 9RT UK; 3 Department of Therapeutic Dentistry, Institute of Dentistry, I. M. Sechenov First Moscow State Medical University, 119146 Moscow, Russia

**Keywords:** CBCT, Maxillary sinus, Sinus membrane, Chronic rhinosinusitis, Odontogenic infection

## Abstract

**Background:**

To assess the change of the Schneider membrane thickness measured by CBCT before and after root canal treatment, retreatment and pulp capping procedures.

**Methods:**

This retrospective study was conducted on CBCT scans of a patient population of Guy’s Hospital NHS Foundation Trust, London. Three groups of patients were studied: Group 1 consisted of patients referred for primary endodontic treatment; Group 2 for endodontic retreatment; Group 3 for indirect pulp capping procedures (serving as a control group). Follow up scans were carried out 1 year after treatment. Measurements were carried out on CBCT scans and data were analysed statistically by Wilcoxon Signed Rank Test. Linear regression was used to assess predictive parameters for membrane thickness.

**Results:**

A statistically significant reduction of the Schneider membrane thickness was observed one year after endodontic treatment and retreatment (*P* < 0.05) but no significant reduction was observed after pulp capping procedures. Linear regression showed that age and gender were significant predictors influencing the Schneider membrane thickness.

**Conclusions:**

Within the limitations of this retrospective study, following root canal treatment and re-treatment a Schneiderian membrane thickness reduction occurred at 1-year follow-up.

The removal of odontogenic infection following endodontic treatment may help reducing the thickness of the Schneider membrane.

## Background

Cone beam computed tomography (CBCT) is a 3D imaging modality that is increasingly used in different fields of dental diagnostics [1]. Adjustments to reduce the radiation dose, artefacts and computational time, while still keeping the detailed image properties, are being researched [2, 3]. With advancements being made, there will be an increase in use of CBCT in the management of endodontic problems [4, 5].

CBCT has also been shown to be more sensitive than conventional radiographs in detecting radiographic signs of endodontic disease such as periapical pathology [6–9].

The operator prescribing the scan has an obligation to report on the entire field of view according to the European Guidelines of Radiation Protection no.136 and 172 [10]. A common incidental finding when taking CBCT scans of maxillary posterior teeth is the thickening of the maxillary sinus membrane immediately adjacent to the apices of these teeth [11, 12].

The sinus is pyramidal shaped with the apex towards the zygomatic process and the base along the nasal wall. The floor of the orbit and the alveolar process of the maxilla are the sides of the pyramid. The maxillary sinus drains via the semi lunar hiatus and the *ostium* into the middle meatus.

The lining of the maxillary sinus is covered by a mucous membrane, the Schneiderian membrane. The membrane is highly vascularised and warms and moistens the incoming air. This ectodermally derived ciliated membrane consists of a pseudo-stratified epithelium with goblet cells, *lamina propria*, and periosteum-like components [13]. It contains large swell bodies, which may become congested when there is an inflammatory or allergic reaction. This leads to thickening of the mucous membrane and is called rhinosinusitis. Acute Rhinosinusitis (ARS) may last up to four weeks. Four or more episodes of ARS within 1 year are referred to as recurrent acute rhinosinusitis. Chronic rhinosinusitis (CRS) refers to those cases which have been symptomatic for at least 12 weeks [14, 15].

Lu et al. (2012) found via CBCT that 48.4% of their subjects had maxillary sinus mucosal thickening, but others reported slightly lower prevalence from 30 to 35% [11, 12, 16]. Twelve per cent of mucosal thickening was found in a large-scale Finnish study, but this analysis was undertaken on panoramic radiographs, which may not be sensitive enough to detect the mucosal thickness [17]. The origin of acute inflammation of the sinus membrane is often odontogenic; the more opacified the sinus is, the more frequently it is related to a dental problem [18, 19].

Pathologic dental findings, root canal treatments, gender, age, and smoking significantly affect the thickness of the sinus membrane according to Vallo et al. [17]. Within the group of dental findings, the degree of marginal and apical periodontitis seemed to be positively associated with the severity of the mucosal thickening of the maxillary sinus (Lu et al. [16]. Implant therapy, mechanical ventilation, extraction and various kinds of trauma by violating the integrity of the bony cavity and the sinus membrane,all introduce the risk for maxillary sinusitis, [15].

The British Association of Otorhinolaryngologists states that 10% of the UK adult population has CRS [19]. Some of these cases may have an odontogenic origin, and therefore may resolve by carrying out root canal treatment [18, 20]. Infected root canal systems may often be the cause of rhinosinusitis, and therefore the aim of this study is to determine whether endodontic treatment and retreatment along with pulp capping procedures have any effect on the thickness of the associated Schneiderian membrane.

The present study focused on the analysis of membrane thickness before and after endodontic/pulp capping procedures with a preoperative and 1-year post-operative follow-up scan.

The null hypothesis was that no difference could be detected between Schneiderian membrane thickness before and after different endodontic and pulp capping procedures.

## Methods

### Subjects

Patients had been recruited as part of broader studies assessing outcomes of indirect pulp capping, primary endodontic and secondary treatment with CBCT. Ethical approval was requested, and granted by the National Research Ethics Committee, London Bridge. Written informed consent was provided by each recruited patient. Exclusion criteria included pregnancy, immunosuppression, unrestorable teeth, and teeth with periodontal probing depths greater than 3 mm, uncontrolled periodontal disease, use of corticosteroids, current or previous smoking, anti-inflammatory and antibiotic together with presence of airways disease or allergy. Whilst the broader studies had recruited patients with teeth requiring treatment from all areas of the mouth, this study only assessed the upper premolars and molars (due to proximity to the sinus) in patients who had returned for their one-year recall.

A total of 138 scans of 69 patients were selected to measure the thickness of the Schneiderian membrane pre- and post-treatment. Sixty upper molars and 14 upper premolars were included in the study. There were three treatment groups, (1) primary endodontic treatment (*n* = 15), (2) endodontic re-treatment (*n* = 36) and (3) indirect pulp capping (*n* = 18). Group 3 served as the control.

### Preoperative examination and radiographic technique

All patients had undergone a preoperative clinical pain history and examination that included assessment of the tooth for tenderness to percussion, mobility and increased probing depths and vitality testing (if appropriate). The soft tissues were assessed for inflammation, the presence of any sinus tracts and tenderness to palpation. A parallel radiograph was taken with a beam-aiming device X-ray unit (Heliodent, Sirona, Bensheim, Germany operating at 65 kVp and 7 mA) and a phosphor plate (Digora® Optime; Soredex, Tuusula, Finland). These patients also had a CBCT scan (3D Accuitomo 80; J Morita, Kyoto, Japan) taken of the area of interest. Each case was scanned preoperatively and at 1-year follow-up. The scanning settings were the following: 90 kVp, 3–5 mA, 17.5 s, 360 basis projections, 40 × 40 mm Field of View, 80 μm voxel size.

### Endodontic procedures

All treatments were performed by specialists or postgraduate trainees of Guy’s and St. Thomas’ Hospital, NHS Foundation Trust, London, UK. The treatments were all carried out under direct supervision of specialist endodontic staff and followed standardised procedures.

### Primary root canal treatment group

This group consisted of patients with teeth in which there was a diagnosis of a carious involvement of the pulp, irreversible pulpitis, acute or chronic apical periodontitis. De novo root canal treatments were completed in one visit by a single operator. Following anesthesia each tooth was debrided from calculus and stripped of existing restorations and following caries removal restorability was confirmed. If required a pre-endodontic restoration was built up with a glass ionomer (Fuji IX glass ionomer cement; GC) to allow rubber dam isolation.

Single use sterile files were used to carry out the procedure. Patency was confirmed with size 8 and 10 k-files (Dentsply Maillefer, Ballaigues, Switzerland). Working length was confirmed with an electronic apex locator (Root ZX II®; J Morita). Canals were enlarged to size 20 to working length prior to rotary shaping with ProTaper Universal instruments (Dentsply Maillefer). Irrigation was carried out with 2% sodium hypochlorite (Chloraxid® 2.0%; PPH Cerkamed, Sandomierska, Poland) throughout the procedure with exchange every 3–4 min, followed by manual agitation with a matching guttapercha point for 30s. A penultimate rinse with 15% EDTA (ENDO-Solution®; PPH Cerkamed) preceded a final irrigation with sodium hypochlorite. The last irrigant was left in the canal and activated with a size 25 Endo-activator® (Denstply Maillefer) for 1 min. Canals were dried with sterile paper points and filled with a warm vertical compaction technique using AH Plus as a sealer (Dentsply Maillefer). Treatment was carried out under microscope magnification and all teeth were referred back for complete cuspal coverage when required.

### Root canal re-treatment group

Subjects participating in this study had been referred to Guy’s and St Thomas’ NHS Foundation Trust, London, UK, for the management of an endodontic problem associated with one or more root filled teeth. Patients were recruited as part of a larger study assessing outcomes of root canal retreatment when comparing CBCT with plain radiographs (ethical approval was requested and granted by the NRES London Bridge and Dulwich Research Ethics Committees). The technical steps were similar to those of the root canal treatment group, removal of gutta-percha was achieved with the use of gates Gliddens, hedstrom files and flexofiles. However, all root canal retreatments were performed over two visits with an inter-appointment calcium hydroxide dressing. All teeth were restored with the appropriate definitive restoration which included full cuspal coverage, if required, provided by the referring dentist.

### Indirect pulp capping group

Teeth included in this group had signs of reversible pulpitis. Clinical examination showed a positive response to vitality testing with endofrost (Coltene, Altstätten, Switzerland) or the Electric pulp tester. Radiographic examination showed deep caries penetrating more than three quarters of the dentine thickness but demonstrated no periapical lesion on CBCT.

Caries removal was carried out under local anaesthetic and rubber dam isolation using a standardised minimal invasive operative protocol. Access through enamel was achieved using a high-speed TA-98 hand-piece (W&H Dentalwerk) with copious irrigation. Superficial soft infected dentine was excavated using carbon-steel rose-head burs (Ash instruments, Dentsply, Gloucester, UK) in a slow-speed WA56A handpiece (W&H Dentalwerk, Bürmoos, Austria) and hand excavator instruments. Deeper caries-infected/affected dentine was removed with the assistance of hand instruments using chemo-mechanical gel (Carisolv™, Rubicon Lifesciences, Gothenburg, Sweden) to aid consistency in caries removal. Any residual caries-affected dentine following chemo-mechanical gel treatment was retained on the pulpal aspect of the cavity. The cavity was then lined using Biodentine (Septodont, Maidstone, UK) or Fuji IX (GC, Tokyo, Japan) following the manufacturers’ instructions. Composite was used as the final restoration following the placement of the pulp capping material.

### Review of patients

Patients were reviewed with a clinical and radiographic examination one year after treatment. During this examination, a post-operative CBCT was taken using the same exposure settings as the initial scan.

For the indirect pulp capping group, the vitality was confirmed at one-year review.

### Radiographic assessment

Two examiners who had not been involved in clinical examination or treatment of patients assessed all the CBCT scans. The examiners were trained using examples of CBCT images with and without the maxillary sinus membrane thickening before carrying out the actual study. Before assessing the experimental material, the reliability of the examiners was assessed by benchmarking, as described previously by Patel et al. 2012. The measurement of maxillary sinus membrane thickening was carried out for 20 CBCT images to determine measurement precision. These images were not from the study subjects.

Two independent standardised observers carried out the measurements for each subject. Measurement spanned the sinus region adjacent to the treated tooth (root canal treatment or re-treatment or pulp capping). The two measurements were carried out on the coronal and sagittal slices at the equatorial level of the tooth of interest. The determination of the area of measurement was determined by tracing a midline on the long axis of the root associated with the maximum membrane thickness from the occlusal aspect of the tooth to the cortical bone limit (including the periodontal ligament or any periapical lesions if present). The actual measurement of the mucosa thickness spanned from the antral side of the mucosa intersected at a right angle to the aforementioned tooth midline as per a previous study [21]. The data for the coronal and sagittal view measurements were averaged.

The CBCT scans were assessed using a Viglen DQ35JO desktop PC (St. Albans, UK) using i-Dixel-3DX version 1.8 software (Morita, Kyoto, Japan), viewed from a Samsung SyncMaster S22B150 1920 × 1080 pixel resolution display, 32-bits colour and the brightness and contrast settings were not modified throughout the assessment.

Intra- and inter-observer reliability was calculated using 20 scans randomly selected and re-measured two weeks after finishing the last measurements.

#### Statistical analysis

Intra-observer agreement was calculated with a Pearson Correlation Test at 95% confidence interval. Satisfactory agreement of the measurements is present for a test value greater than 0.8. Data were pre-assessed and checked for normality with the Kolmogorov-Smirnov and the Shapiro-Wilk tests. The required sample size was determined using a power calculation based on the Altman’s nomogram at 80% level of power. A previous study (Nurbakhsh et al. 2011) reported a clinically relevant difference for membrane thickness over 3.59 mm.

Comparison of the thickness of the Schneiderian membrane pre and post endodontic treatment was performed for each subject using a non-parametric test (Wilcoxon Signed Rank), with a significance level set at 0.05. A McNemar’s test was used to determine associations between the reduction of the Schneiderian membrane thickness and the periapical healing observed with the CBCT imaging at 12 months. Linear regression analysis was used to determine the effect of age and gender on membrane thickness. The statistical software package used was SPSS 22 (IBM, Armonk, USA).

## Results

Sample size determination revealed a minimal number of 15 patients, for each of the three groups, necessary to achieve a power of 80% at a significance level of 0.05. Patients’ characteristics distribution is shown in Table 1.

**Table 1:** Population demographics

Group	Mean age	Age range	Gender	
			Female	Male
Root canal treatment	54.7	38–75	11	4
Root canal retreatment	43.5	16–85	25	11
Indirect pulp capping	38.1	24–78	6	12

Mean age, age range and gender division among the observational groups

Pearson’s Correlation test revealed a good intra- and inter-observer agreement (0.98 and 0.92 respectively) for the repeated measurements of the membrane thickness.

As per the Kolmogorov-Smirnov and the Shapiro-Wilk tests the data regarding the sinus membrane thickness were non-normally distributed, therefore non-parametric tests were selected.

**Fig. 1:**
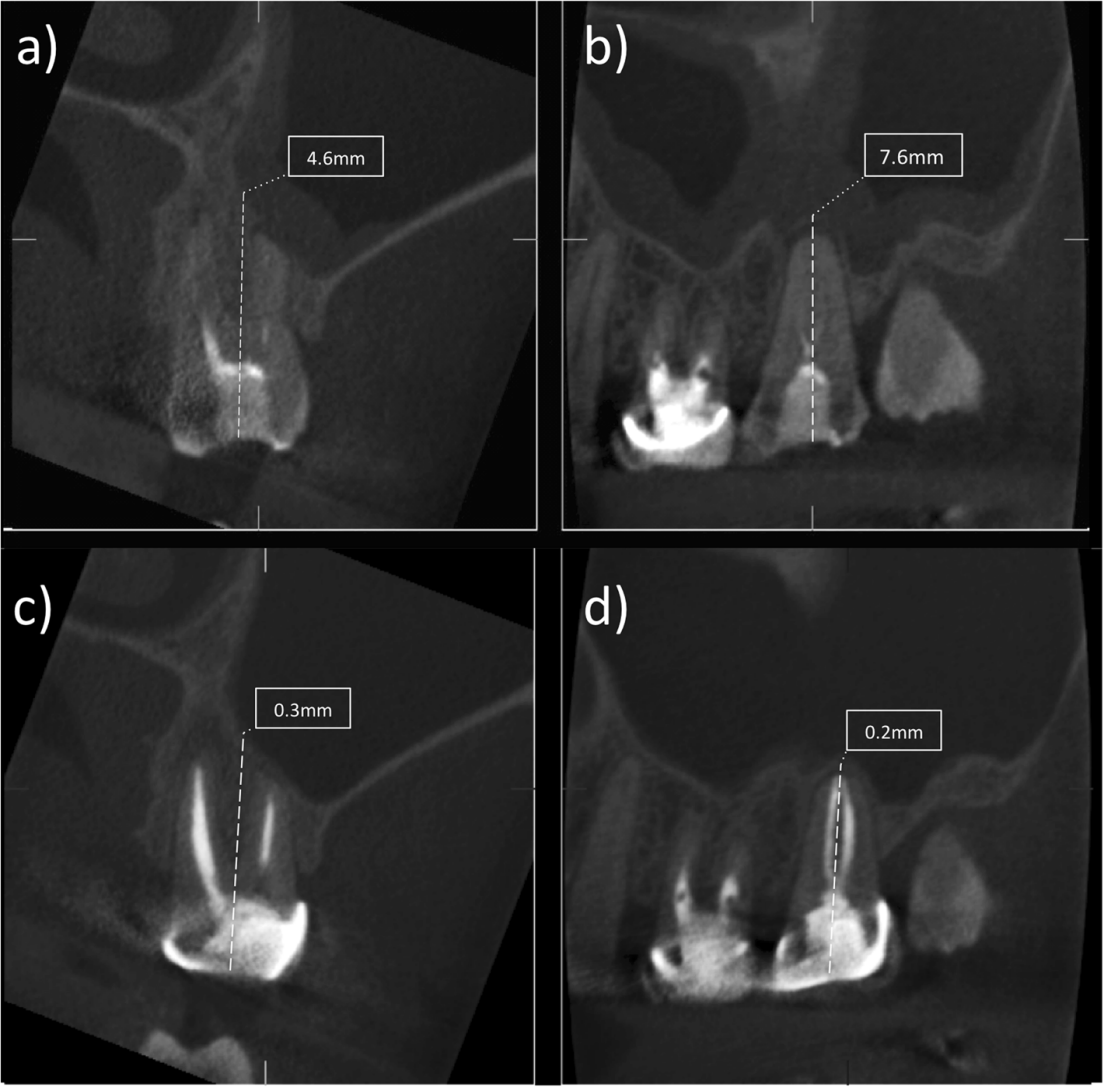
(a-d). Image showing the pre- and post treatment measurements on the coronal (a,c) and sagittal cone beam computed tomography slices (b,d) related to an upper left second molar area with the relative measurement of the Schneider membrane thickness in mm before root canal re-treatment (a,b) and after one year follow-up (c,d)

**Fig. 2:**
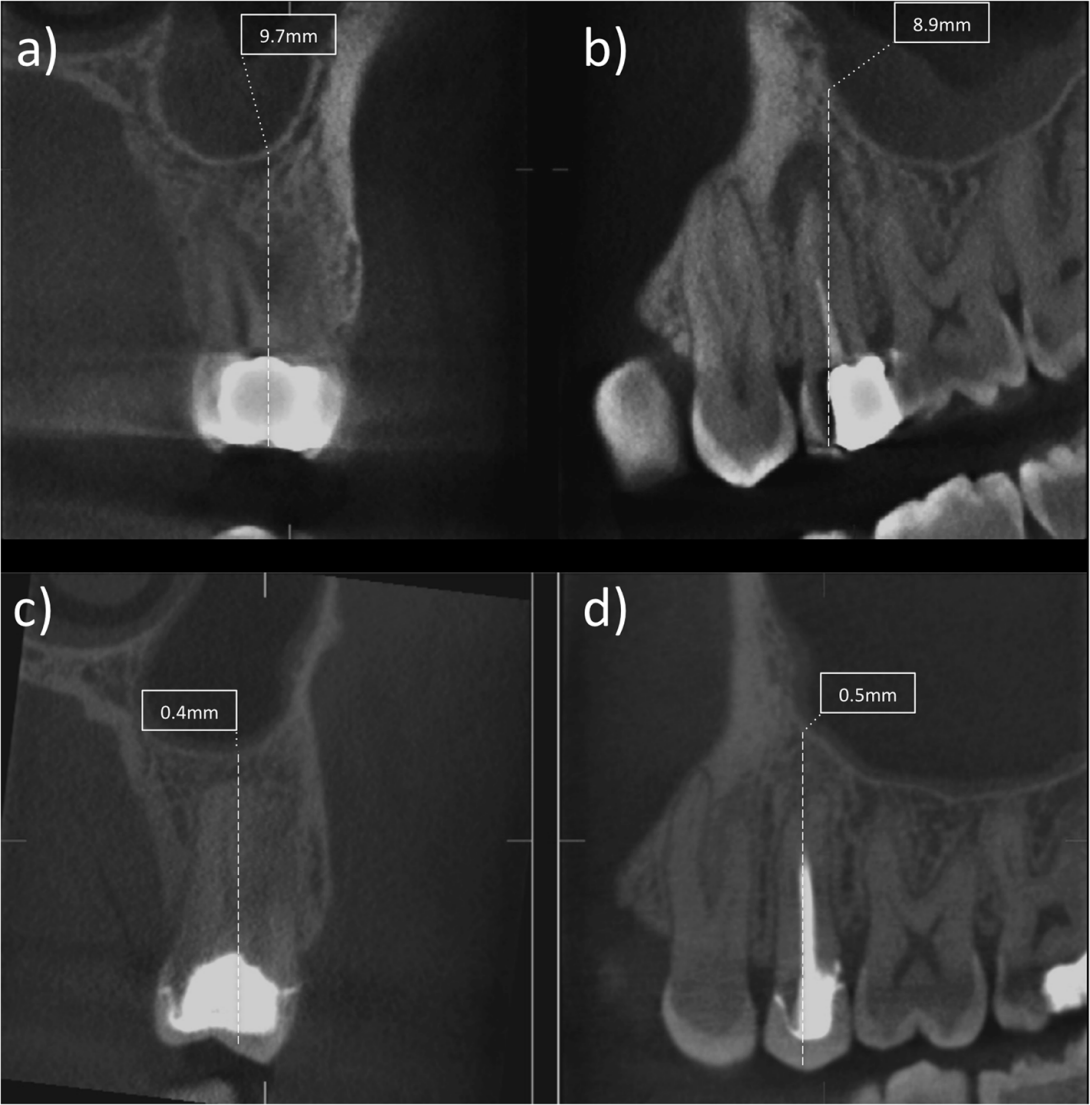
(a-d). Image showing the pre- and post treatment measurements on the coronal (a,c) and sagittal cone beam computed tomography slice (b,d) related to an upper left second molar area with the relative measurement of the Schneider membrane thickness in mm before root canal re-treatment (a,b) and after one year follow-up (c,d)

Comparison of the means revealed a statistically significant difference between pre- and post-treatment Schneiderian membrane thickness measured in endodontically treated and re-treated teeth (*P* < 0.05): with a smaller mean membrane thickness at 1 year after treatment (mean: 8.2 mm vs. 4.3 mm) and re-treatment (mean: 7.4 mm vs 2.5 mm) (Figs. 1 and 2) (Table 2). There was no statistically significant difference between primary and secondary treatment in terms of magnitude of change of the Schneider membrane thickness. There was a significant association (McNemar test, *P* = .035) between the reduction of Schneiderian membrane thickness at 12 months and periapical healing at 12 months.

**Table 2:** Descriptive statistic for each measured group

Group	Mean	Median	Maximum	Minimum	95% CI
1 (pre-op)	8.2	6.9	19.4	0	5.1–11.36
1 (post-op)	4.3	3.9	13.8	0	3.1–6.5
2 (pre-op)	7.4	5.8	19.5	1.04	3.9–9.9
2 (post-op)	2.7	2.0	13.2	0	1.7–3.7
3 (pre-op)	5.1	4.8	14.4	0.88	1.9–6.7
3 (post-op)	3.8	2.1	10.2	0	1.5–5.9

Mean, Median Maximum, minimum and confidence interval for Schneider membrane thickness in mm

1) Root canal treatment; 2) Root canal re-treatment; 3) Indirect pulp capping

In particular, with respect to the apical lesion size in the treatment group, mean of reduction was − 1.85 ± 0.71 mm, and − 2.23 ± 1.35 mm in the retreatment group. Comparison of the mean membrane thickness for pulp capping cases revealed no statistically significant differences at one-year recall (mean: 5.1 mm vs. 3.8 mm) (*P* > 0.05).

Linear regression analysis showed that age had a significant predictive value, and with increase of age there was also an overall increase of the mucosal thickness. With respect to gender the analysis showed that male subjects had a less thick maxillary sinus membrane.

## Discussion

In a recent cross-sectional study, the mean thickness of the maxillary sinus membrane varied between 2.1 and 2.69 mm as per CBCT imaging. Only 35% of the evaluated sinuses exhibited a healthy mucosa. Gender did not influence the thickness of the sinus membrane at the root tips of the premolars or at single-tooth gaps, but there was a statistically significant correlation in the region of the maxillary molars.

A recent study failed to determine a correlation with mucositis of the antrum and periapical healing, however the follow-up time was limited to 3 months [22].

The distinction between allergic reaction and thickening due to anatomical or inflammatory issues should be determined and this could be achieved by using larger field of view scans. To look at enlargement of the posterior tip of the inferior turbinate as a sign of allergic response was suggested [23]. However, because of the small FOV, used in this study, according to the ALARA-principles and region of interested scanned, such measurements would not be feasible.

Vallo et al. (2010) compared the mucosal thickness in subjects with and without pathological dental findings to totally edentulous subjects. Dentate subjects with pathological findings had a higher risk of having mucosal thickening. Moreover, edentulous subjects did not differ from subjects who had no dental pathology. The age did influence different presentation of the mucosal thickening with a higher prevalence of mucosal antral cysts in young patients below 40 years old, whereas a more prevalent mucosal thickening was observed in the 40–49 years old group. Due to lack of availability of CBCT’s from such groups of patients and the age dispersion, we used the pulp-capped teeth group as the control. They had no signs and symptoms of apical problems and showed no change in thickness [17]. Therefore, pulp-capped teeth, with possibly limited level of inflammation and infection proved as an appropriate control group: in facts current findings seem to point to the advantages of taking a pre-operative CBCT scan in pulpotomy cases to rule out the presence of pre-existing thickening of the Schneider membrane and /or periapical lesions in order to maximise the success [24]. Alternatively, as negative control, of the healthy Schneider membrane thickness, virgin teeth could have been selected, however the presence of potential neighbouring swelling close to the affected teeth suggested to discard teeth adjacent to the treated and re-treated ones. On the other hand, selecting non treated sites was not recommended due to the ALARA principles as this would have required a CBCT scan of healthy teeth with no suspected pathology. Similarly, the choice of wide FOV CBCT scans would have unnecessarily exposed the healthy contralateral sinus and reduced the radiographic resolution of the area of interest. As per current Endodontic guidelines a small FOV scan is recommended [25].

Lu et al. found that patients over the age of 60 were most likely to present with sinus mucosal thickening [16]. Conversely in a European epidemiological study the prevalence of CRS was lower in older than in younger subjects, therefore, the symptoms might not correlate with the actual presence of CRS [26].

In the present cohort there were more female than male subjects both in the retreatment (25 vs. 11) and treatment group (11 vs. 4), whereas in the pulp capped group there were more male than female (12 vs. 6) subjects. The linear regression showed that there was a correlation of female gender on increased thickness of the Schneiderian membrane; therefore, a certain bias may have derived from the uneven distribution of genders among groups. However, in retrospective studies these factors are not controllable. Furthermore, due to the lack of randomisation present in retrospective studies any causal inference regarding the gender and age on the Schneiderian membrane thickening should be avoided. However, all the above-mentioned confounding factors would certainly equally affect the pre-operative and post-operative thickness of the membrane; therefore, the variation of the thickness should be largely free of biases. In elderly patients a pre-existing thickening of the Schneider membrane may reduce the magnitude of the post-operative thinning of the membrane. Further studies should involve a wider randomised cohort of patients and involve a sub-group analysis focused on the age relationship to the membrane thickness before and after treatment.

Another factor that might have acted as a confounder on the membrane thickness was the presence of multiple teeth treated in the area of interest. Both in the treatment and re-treatment group two patients had more than one tooth treated at once. An addictive effect of the odontogenic infection on the Schneiderian membrane thickness may occur in such cases. Previous work however has shown no link between the endodontic status of adjacent teeth and antral mucosal thickness [27].

Independently of the potential confounders, a significant reduction of the Schneider membrane thickness was detected following primary and secondary endodontic treatment. Furthermore a positive correlation was established between the thinning of the Schneider membrane and the reduction of the periapical bone lesions following periapical healing. The underlying pathological mechanisms behind the thickening of the antral mucosa in the presence of endodontic pathology are elucidated by the only in vivo study available, where odontogenic sinusitis was induced, and histological and microbiological analysis confirmed the biological processes involved. In particular the histology revealed mucosal oedema, with infiltration of granulocytes and lymphocytes together with squamous cell metaplasia and areas of desquamated epithelium. These aspects indicate a typical cytokine cascade induced by the presence of endodontic infection with inflammation mediators recruitment and diffusion to the periapical area [28].

The changes of thickness of the Schneider membrane are clinically relevant to the Endodontic specialists at the time of diagnosis and to assess the signs of healing od endodontic infection. Furthermore, Otorhinolaryngology specialists may point the patients to the odontogenic origin of certain mucosal antral cysts.

The one-year follow-up showed a significant reduction of the Schneiderian membrane thickness following root canal treatment and re-treatment. To date this is first study determining changes in the maxillary mucosa membrane thickness in paired samples.

Future studies should address whether failure of root canal treatment and retreatment would cause a persistence of the Schneiderian membrane thickening, however a very large sampling number would be required to achieve the power necessary to discern such occurrence in failed cases.

## Conclusions

Within the limitation of the present retrospective study it can be inferred that endodontic treatment and re-treatment were effective in reducing the thickness of the Schneiderian membrane.

The pulp capped group showed no significant difference in membrane thickness before and after treatment.

The relationship between thickness, pathology and behaviour of the Schneiderian membrane and endodontic infection should be addressed using prospective clinical investigations.

## Data Availability

The datasets used and/or analysed during the current study are available from the corresponding author on reasonable request.

## Abbreviations

CBCT: Cone beam computed tomography
EDTA: Ethylenediaminetetraacetic acid
NHS: National Health Service
ARS: Acute Rhinosinusitis
